# A particular appearance of a diaphragm pannus completely masking a mechanical mitral valve: a case report

**DOI:** 10.11604/pamj.2025.50.115.47267

**Published:** 2025-04-30

**Authors:** Yassine Morjane, Manal Msirdi, Zakaria Bazid, Nabila Ismaili, Noha Elouafi, Hicham El Malki, El Mehdi Moutaouekkil

**Affiliations:** 1 Department of Cardiovascular Surgery, Mohammed VI University Hospital Center, Oujda, Morocco,; 2 Department of Cardiology, Mohammed VI University Hospital Center, Oujda, Morocco,; 3 Faculty of Medicine and Pharmacy, Mohammed the First University, Oujda, Morocco

**Keywords:** Pannus, mechanical valve prosthetic, echocardiography, cinefluoroscopy, case report

## Abstract

In this article, we report the case of a 58-year-old man who was admitted to our institution with acute dyspnea and poor hemodynamic condition. He had undergone double mitral-aortic valve replacement five years earlier for rheumatic heart disease. Transthoracic echocardiography (TTE), cinefluoroscopy, and transesophageal echocardiography (TEE) revealed a significant reduction in the opening of the mechanical mitral valve leaflets. An urgent redo surgery was performed, during which the pannus was removed, ensuring the restoration of normal leaflet motion. This clinical case presents two rare circumstances: first, the unusual diaphragmatic pannus completely masking the mechanical mitral prosthetic valve, and second, the development of pannus at the level of the mitral mechanical prosthesis rather than the aortic mechanical prosthesis.

## Introduction

Mechanical valve prostheses have the advantage of a long service life, with thrombosis and pannus being the main causes of malfunction, potentially leading to prosthetic blockage [1,2]. Thrombotic complications are more common in the early postoperative period, whereas pannus typically develops later [3]. We report a case of bileaflet mechanical valve failure in the mitral position due to an unusual pannus formation, necessitating reoperation.

## Patient and observation

**Patient information:** a 58-year-old man with no cardiovascular risk factors underwent double heart valve replacement for severe rheumatic mitral and aortic stenosis using 31mm and 21mm Carbomedics mechanical prosthetic heart valves.

**Timeline of current episode:** five years later, the patient was admitted to the cardiology department with acute dyspnea and hemodynamic instability, with no reported history of fever or chest pain.

**Figure 1:**
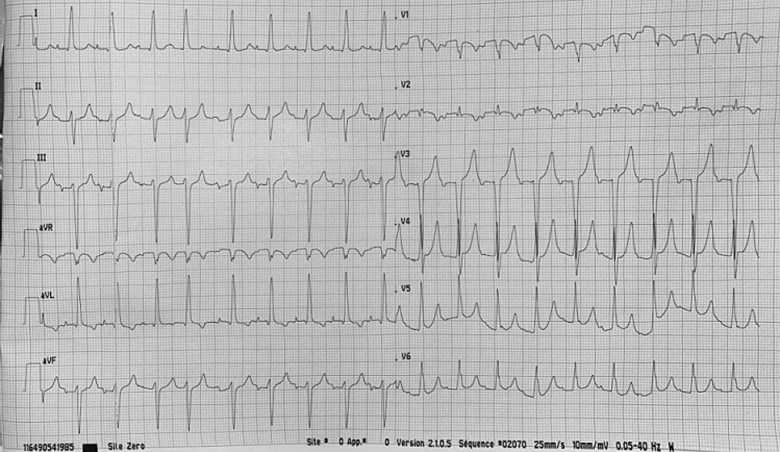
electrocardiogram at admission

**Clinical findings:** physical examination revealed shortness of breath, polypnea, a decrease in oxygen saturation to 80%, tachycardia, and hypotension. Cardiac auscultation showed diminished closing clicks of the mitral prosthetic valve, while pulmonary auscultation revealed crackling rales at the apices. The electrocardiogram demonstrated atrial tachyarrhythmia at 150 beats per minute (Figure 1).

**Figure 2:**
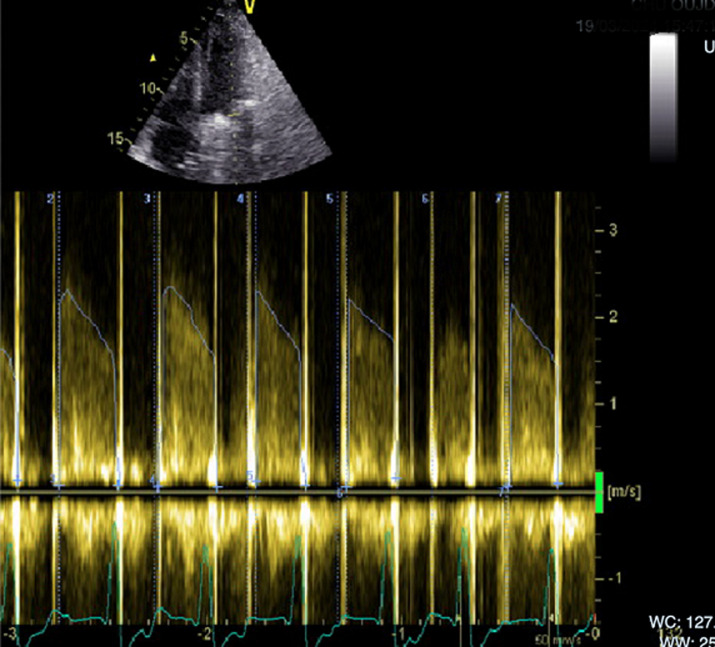
increased gradient across the mitral prosthetic valve

**Figure 3:**
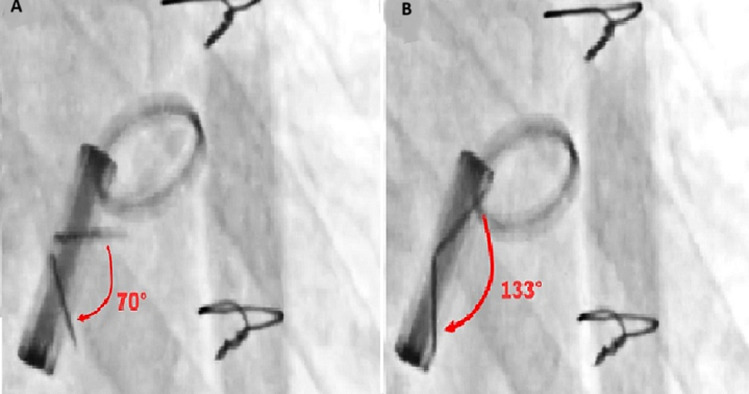
cinefluoroscopy: A) diastolic image showing both mitral discs not fully open, with an opening angle of 70° (normal value for the Carbomedics mitral bileaflet mechanical prosthetic valve is <24°); B) systolic image showing a closing angle of 133° (normal value for the Carbomedics mitral bileaflet mechanical prosthetic valve is >130°)

**Diagnostic assessment:** biological examinations revealed a hemoglobin level of 12.2 g/dL, a biological inflammatory syndrome with a C-reactive protein level of 264 mg/L, and neutrophil-predominant hyperleukocytosis of 18,500/mm^3^, while renal and liver function tests were within normal limits. Transthoracic echocardiography showed a blocked leaflet of the mitral prosthetic valve without any thrombus image on either the ventricular or atrial side, while the aortic prosthetic valve function remained normal. The mitral trans-prosthetic pressure gradient was significantly elevated, with a mean gradient of 18.09 mmHg (Figure 2). The effective mitral orifice area, calculated using the continuity equation, was 0.8 cm^2^, and the Doppler velocity index was 3.3, all indicating significant obstruction of the mitral prosthesis. The left ventricle was not hypertrophied and had preserved global function (ejection fraction of 57%), whereas the right ventricle was dysfunctional with severe pulmonary hypertension. To assess the motion of the prosthetic leaflets, cinefluoroscopy was performed (Figure 3). It revealed a limited opening of the mitral prosthetic leaflets, with a diastolic opening angle of 70°, compared to the normal value of less than 24° for a Carbomedics mitral bileaflet mechanical prosthesis. The systolic closure of the leaflets remained preserved, with a closing angle of 133° (the normal value for the Carbomedics mitral bileaflet mechanical prosthesis is >130°).

**Diagnosis:** three main diagnoses were considered: a mitral prosthetic thrombus, which was deemed less likely due to the absence of thrombus visualization on echocardiography; infective endocarditis, which remained a possibility given the biological inflammatory syndrome. The patient was considered at high risk for endocarditis despite the absence of other minor or major modified Duke diagnostic criteria; and finally, the presence of a pannus formation, which was highly suggestive due to the five-year interval before symptom onset.

**Figure 4:**
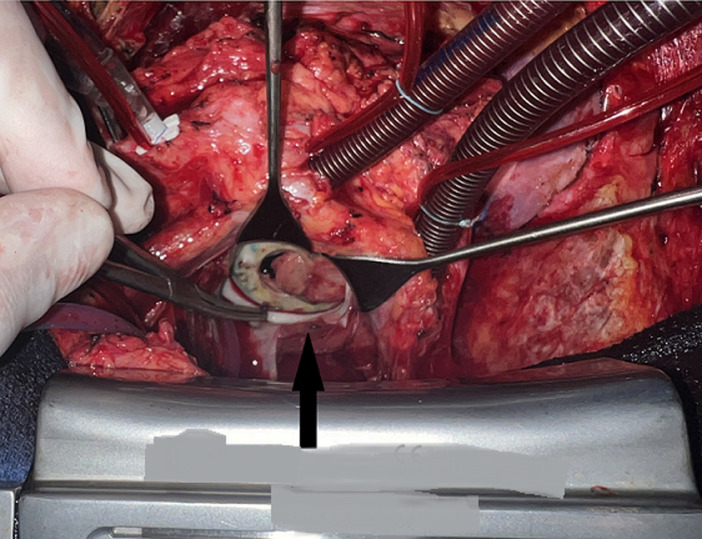
intraoperative view showing an unusual diaphragmatic pannus completely obscuring a mechanical mitral prosthetic valve

**Figure 5:**
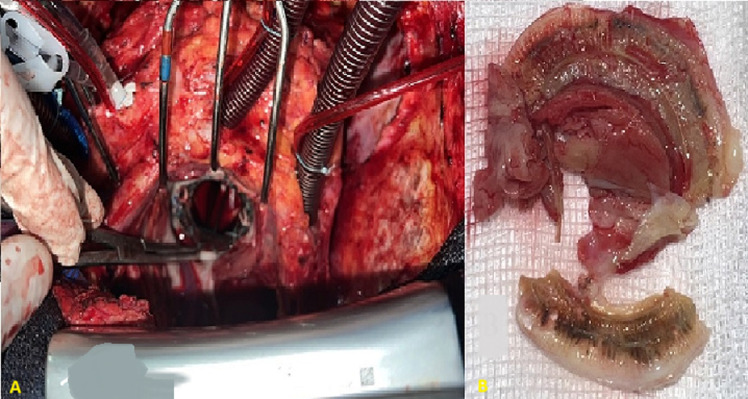
A,B) intraoperative view showing complete pannus resection

**Therapeutic interventions:** the acute pulmonary edema caused by the complete blockage of one of the two prosthetic leaflets in our patient represented a surgical emergency. The patient was placed on oxygen, inotropes, and furosemide, but showed no clinical improvement, necessitating surgery. A median redo sternotomy was performed, and conventional cardiopulmonary bypass was established. Myocardial protection was achieved with a single dose of custodial after cross-clamping. A left atriotomy was then carried out, revealing a left atrium completely free of thrombosis. Inspection showed a highly unusual diaphragmatic pannus completely covering both leaflets, almost entirely restricting their opening (Figure 4). A complete resection of the pannus was performed, restoring normal leaflet function (Figure 5).

**Follow-up and outcome of interventions:** postoperative TEE assessment of the mitral and aortic prosthetic valves was satisfactory, and the patient was transferred to the intensive care unit. The postoperative recovery was uneventful.

**Patient perspective:** the patient expressed optimism for a full recovery.

**Informed consent:** the patient provided written consent for the publication of his clinical details and any identifying images.

## Discussion

A number of complications can arise after valve replacement surgery, some of which are common to all cardiac surgeries, while others are specifically related to prosthetic valves. Blockage of a prosthetic valve is a rare but potentially fatal complication [1,2]. The main causes are thrombosis and pannus formation around the prosthesis, with the latter typically occurring later after surgery [3]. Pannus is a fibrous tissue that develops around cardiac prostheses, usually in an annular location, as a response to blood stasis and local inflammation [4]. Its development primarily depends on patient-related factors, the type of prosthesis, its design, size, and location. Pannus is more commonly observed on aortic prostheses than mitral ones, and when it occurs in the mitral position, it is usually located on the atrial side of the prosthesis. Additionally, the presence of pannus can predispose the valve to thrombosis, while chronic thrombosis of a cardiac prosthesis can also trigger pannus formation [4,5]. Symptoms vary depending on the severity of prosthetic dysfunction [1-6]. The incidence of such dysfunction in cardiac prostheses is reported to range between 0.1% and 6% per year [7], and in most cases, it occurs several years after valve surgery. However, valve dysfunction due to early pannus formation has been repeatedly documented, with the first cases diagnosed between 1 and 12 months post-surgery. Shi *et al*. reported a rare case of early pannus formation on a mitral prosthesis just three months after surgery [8], while Cleveland *et al*. described a case occurring one month after aortic valve replacement [9].

The exact mechanism behind excessive pannus formation in some patients remains unclear, but it has been linked to various factors, including rheumatic fever, atrial fibrillation, low cardiac output, high transprosthetic gradients, small valve size, and ineffective anticoagulation [5]. This article highlights a rare circumstance: the development of pannus on the mitral mechanical prosthesis rather than on the aortic mechanical prosthesis, despite both valves being replaced in the same operation. A possible explanation could be left atrial dilation, inadequate anticoagulation, and atrial fibrillation, which may promote pannus formation and obstruction even with a larger-diameter mitral prosthesis. In contrast, the high hemodynamic stress and large vascular bed distance in the aortic position may have provided a protective effect against pannus formation. Clinical diagnosis and confirmation through imaging remain challenging. The most effective diagnostic tools include TTE, TEE, fluoroscopy, and axial computed tomography. The therapeutic approach depends on several factors, including the severity of valve obstruction, the patient's clinical condition, and the prosthesis location. Redo surgery is recommended in cases of critically symptomatic mechanical valve obstruction [1-6]. In our case, the patient presented with acute pulmonary edema due to complete blockage of one of the two leaflets. Despite oxygen therapy and medical treatment with inotropes and furosemide, there was no clinical improvement. Given the critical situation, a heart team discussion led to the decision for reoperation. We strongly suspected that the obstruction was primarily due to pannus formation, which was subsequently confirmed intraoperatively.

## Conclusion

We would like to draw the doctor's attention to two rare aspects of our clinical case: first, the unusual diaphragmatic pannus completely obscuring a mechanical mitral prosthetic valve, and second the development of pannus on the mitral mechanical prosthesis rather than on the aortic mechanical prosthesis, despite both valves being replaced in the same operation.
